# Supplementary material for: Cellulose Paper Modified by a Zinc Oxide Nanosheet Using a ZnCl2-Urea Eutectic Solvent for Novel Applications
Source: Nanomaterials (Basel). 2021 Apr 25;11(5):1111. doi: 10.3390/nano11051111 (PMC8146088; doi:10.3390/nano11051111)
Supplement: Supplementary file 1 [file nanomaterials-11-01111-s001.zip › nanomaterials-1193406-supplementary.pdf]

nanomaterials

MDPI

Supporting information

Cellulose Paper Modified by Zinc Oxide Nanosheet Using a $ZnCl_2$-Urea Eutectic Solvent for Novel Applications

Changmei Lin [1], Duo Chen [2], Zifeng Hua [1], Jun Wang [1], Shilin Cao [1,*], and Xiaojuan Ma [1,*]

[1] College of Materials Engineering, Fujian Agriculture and Forestry University, Fuzhou 350002, China; 15980205608@163.com (C.L.); 13459735703@163.com (Z.H.); wangjun20201118@163.com (J.W.)
[2] International School for Optoelectronic Engineering, Qilu University of Technology (Shandong Academy of Science), Jinan 250353, China; sdkdcd@163.com
* Correspondence: fafucsl@fafu.edu.cn (S.C.); 000q151067@fafu.edu.cn (X.M.)

Table S1. The ZnO content of different ZnO@paper.

Sample	Blank paper	ZnO@paper-1	ZnO@paper-2	ZnO@paper-3	ZnO@paper-4	ZnO@paper-5
ZnO/wt%	0	8.0	10.0	12.7	16.3	20.3

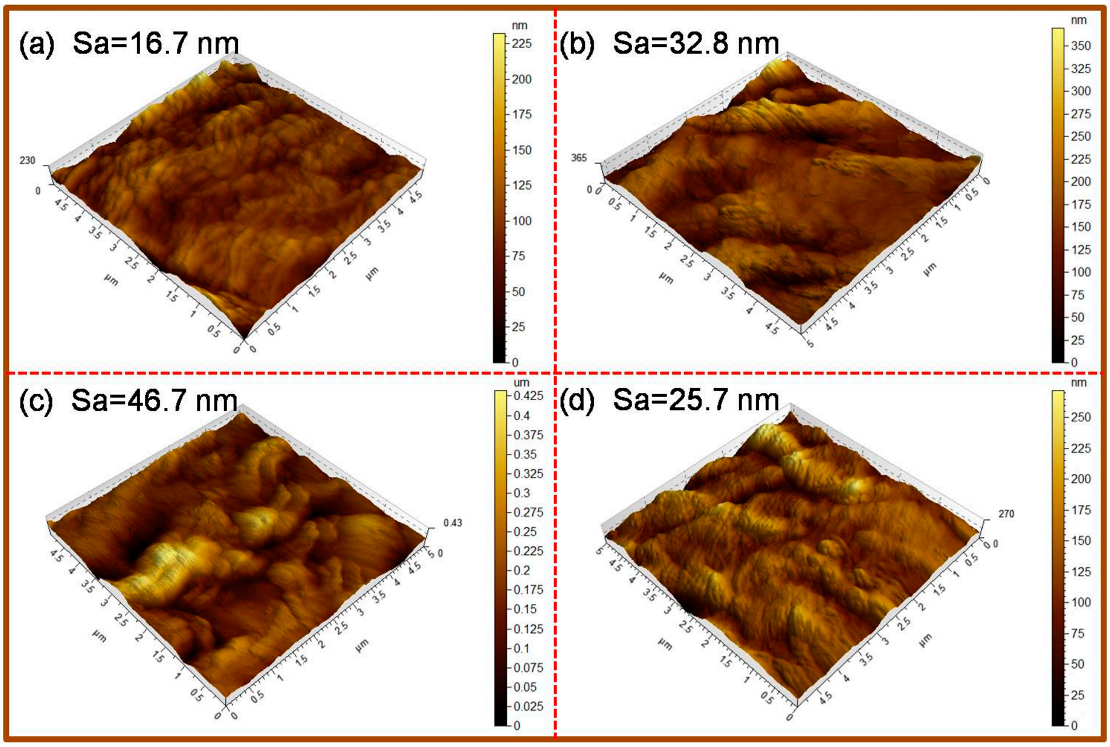

Figure S1. AFM image of **(a)** blank paper, **(b)** ZnO@paper-2, **(c)** ZnO@paper-4, **(d)** ZnO@paper-5.

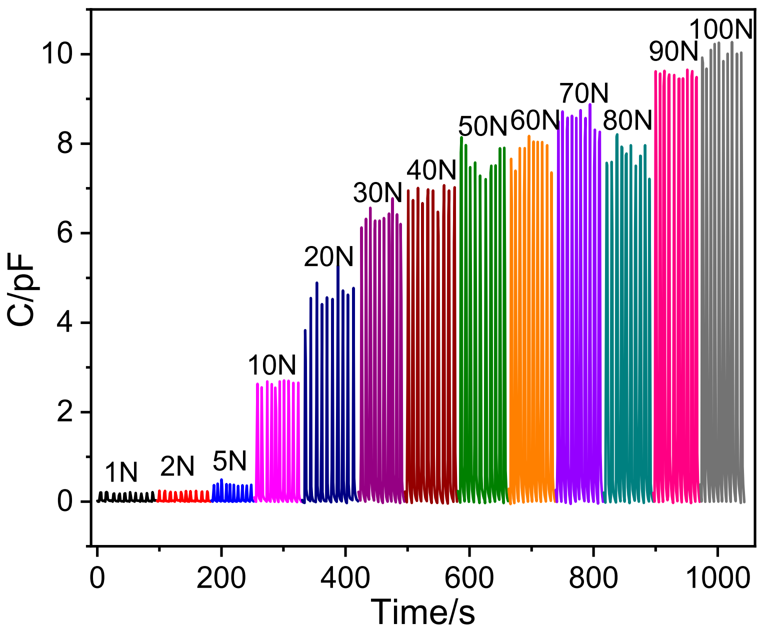

Figure S2. The output signal of the pressure sensor in response to applied pressure.
